# The p38α mitogen-activated protein kinase as a central nervous system drug discovery target

**DOI:** 10.1186/1471-2202-9-S2-S12

**Published:** 2008-12-03

**Authors:** Aaron S Borders, Lucia de Almeida, Linda J Van Eldik, D Martin Watterson

**Affiliations:** 1 Center for Drug Discovery and Chemical Biology, Northwestern University, Chicago, IL 60611, USA; 2 Department of Cell and Molecular Biology, Northwestern University, Chicago, IL 60611, USA; 3 Department of Molecular Pharmacology and Biological Chemistry, Northwestern University, Chicago, IL 60611, USA

## Abstract

Protein kinases are critical modulators of a variety of cellular signal transduction pathways, and abnormal phosphorylation events can be a cause or contributor to disease progression in a variety of disorders. This has led to the emergence of protein kinases as an important new class of drug targets for small molecule therapeutics. A serine/threonine protein kinase, p38α mitogen-activated protein kinase (MAPK), is an established therapeutic target for peripheral inflammatory disorders because of its critical role in regulation of proinflammatory cytokine production. There is increasing evidence that p38α MAPK is also an important regulator of proinflammatory cytokine levels in the central nervous system, raising the possibility that the kinase may be a drug discovery target for central nervous system disorders where cytokine overproduction contributes to disease progression. Development of bioavailable, central nervous system-penetrant p38α MAPK inhibitors provides the required foundation for drug discovery campaigns targeting p38α MAPK in neurodegenerative disorders.

## Background

Mitogen-activated protein kinases (MAPKs) are a family of serine/threonine protein kinases that play essential roles in eukaryotic cells by transducing environmental stress signals into altered gene expression. There are numerous human MAPKs, which are grouped into distinct families: the extracellular signal-regulated protein kinases (ERKs); the c-Jun N-terminal kinases (JNKs); and the p38 MAPKs (p38α, p38β, p38δ, p38γ). Different stressors, or combinations of stressors, result in differential activation of the discrete MAPK families, which can function in parallel in intracellular signal transduction cascades that alter cellular physiology. Signaling cross-talk among the individual MAPK cascades, as well as cross-talk with second messenger-mediated protein phosphorylation cascades, result in a high degree of biological selectivity in a tissue's response to stressors. Therefore, the presence of a given MAPK family member in a tissue or cell type does not provide a simple forecast of its physiological or pathophysiological role.

Various genetic and pharmacological inhibitors of individual protein kinases in stress-activated cells have provided causal linkages between the activation of a given kinase, or MAPK family pathway, and a particular cellular response endpoint, for example, increased production of proinflammatory cytokines. The p38 MAPK family of serine/threonine protein kinases was explicitly implicated in the regulation of key inflammatory responses in mammals, contributing to a large body of evidence that eventually established it as a therapeutic target for a range of diseases that have inflammation as a common disease progression mechanism. An isoform of the p38 MAPK family, p38α MAPK, was identified early as a drug discovery target and became the focus of intense investigations for over a decade. Currently, novel p38α MAPK inhibitors are in clinical development for peripheral tissue inflammatory disorders. On-going investigations continue to validate p38α MAPK as a therapeutic target for peripheral tissue disorders, displaying no target-related toxicities when appropriate compounds and dosing regimens are used. However, *in vivo *evidence supporting p38α MAPK as a central nervous system (CNS) therapeutic target has only recently become available. Here we provide a brief review of these emerging CNS data and highlight selected work that provided the firm foundation for considering bioavailable, blood brain barrier-penetrant, non-toxic p38α MAPK inhibitors as potential therapeutics for CNS disorders.

## The p38 MAPK family as regulators of proinflammatory cytokine production

Proinflammatory cytokines are crucial components of physiological defense mechanisms, but chronic overproduction can lead to cellular dysfunction and damage [1]. One pathophysiology mechanism for peripheral tissue injury is the overproduction of proinflammatory cytokines, for example, tumor necrosis factor (TNF)α and interleukin (IL-1)β, which can lead to tissue barrier dysfunction and cell death. Current macromolecular therapeutics for peripheral tissue disorders used in the clinic target this increased cytokine activity [1]. Intracellular MAPK signal transduction cascades, especially the p38 MAPKs, are important regulators of proinflammatory cytokine biosynthesis [2-4]. p38 MAPK was first identified as a key regulator of IL-1β and TNFα production in human monocytes after lipopolysaccharide treatment [5,6]. Later studies showed that activation of p38 MAPK regulates proinflammatory cytokine production at the transcriptional and post-transcriptional levels [7,8], laying the foundation for exploration of p38 MAPK as a potential drug discovery target for attenuation of increased proinflammatory cytokine levels [3,4].

Four isoforms of p38 MAPK have been identified, each the product of distinct genes: p38α, 38β, p38γ and p38δ [2,9]. There are also several splice variants of these isoforms. p38α MAPK is widely expressed among tissues and is considered a crucial mediator of inflammatory responses activated by a variety of signaling mechanisms with a wide range of physiological endpoints [6,10,11]. Recently, O'Keefe *et al*. [12] demonstrated in an elegant approach using knock-in mice that the specific inhibition of the p38α isoform *in vivo *is sufficient and necessary for suppression of increased peripheral proinflammatory cytokine levels after lipopolysaccharide challenge. As with many intracellular signaling cascades mediated by serial protein phosphorylation steps, p38α MAPK is activated via transphosphorylations by upstream kinases [2]. The activation of p38α MAPK, in turn, allows it to efficiently phosphorylate its protein substrates [13]. The exact physiological outcomes from such integrated, complex networks are dependent on the type of stressor, cell type, tissue context of the cell, and previous stimulations.

In terms of the regulatory mechanisms of proinflammatory cytokine production, several of the p38 MAPK substrates are transcription factors, or other protein kinases, which in turn can phosphorylate regulatory proteins and thereby modulate function [13]. For example, p38α MAPK can phosphorylate a variety of transcription factors, for example, ATF2, ELK1, CREB, MEF2C, CHOP/GADD153, and C/EBPβ, leading to transcriptional stimulation of proinflammatory cytokines [14]. There are also a variety of p38α MAPK substrates that can regulate proinflammatory cytokine production through either transcriptional or translational mechanisms. One of the first endogenous substrates identified for p38α MAPK was MAP kinase-activated protein kinase-2 (MAPKAP K2 or MK2), which is critical for the biosynthesis of TNFα after lipopolysaccharide treatment [15-17]. This pathway has also been proposed to stabilize cytokine mRNA by mechanisms dependent on AU-rich elements in the untranslated regions of the cytokine genes [18,19]. Another protein kinase substrate of p38α MAPK is mitogen and stress activated protein kinase 1 (MSK1), which is activated upon phosphorylation by p38α MAPK. Activated MSK1 can, in turn, stimulate transcription factors, allowing increased proinflammatory cytokine production [20]. MSK1 also appears to be involved in the expression of proinflammatory cytokine genes through phosphorylation of histone 3 (H3) and recruitment of NFκB [21-23]. The p38 MAPK/MSK1 pathway is also important for phosphorylation of CREB, a regulatory protein implicated in proinflammatory cytokine gene expression [24,25]. Consistent with these proposed biological roles in cell function, p38 MAPKs are found in both the nucleus and cytoplasm [14,26]. Thus, the wide variety of downstream substrates of p38 MAPKs, along with the spectrum of stressors and upstream activators that can converge on p38 MAPK activation, allow for fine control of proinflammatory cytokine production. The pivotal role of these pathways in the regulation of responses resulting in increased proinflammatory cytokine activity emphasizes the detrimental consequences dysregulation of these kinase cascades can have in disease.

## Modulation of p38α MAPK as a therapeutic approach to peripheral inflammatory disorders

p38α MAPK is an established drug discovery target for peripheral inflammatory diseases, including rheumatoid arthritis and Crohn's disease, where increased levels of proinflammatory cytokines coincide with disease progression [27-30]. Treating animal models of these diseases with p38α MAPK inhibitors reduces the expression of proinflammatory cytokines, for example, IL-1β and TNFα, and alters disease-related pathology [31-33]. Early clinical development studies for the treatment of peripheral inflammatory diseases revealed toxicity issues related to the chemotype of the inhibitor and not to the target [34,35]. This knowledge was used in the recursive development of later generation inhibitors based on structurally distinct chemotypes that showed improved safety, metabolic stability, and pharmacokinetic profiles while retaining or improving upon the affinity and selectivity for p38α MAPK [36-38].

Currently, p38α MAPK inhibitors based on a variety of chemotypes are in clinical development for the treatment of peripheral tissue diseases, for example, multiple myeloma, atherosclerosis, chronic obstructive pulmonary disease, rheumatoid arthritis, and pain. Compounds include, among others, SCIO 469 (Scios, Johnson and Johnson), VX-702 (Vertex), and SB 681323 (GlaxoSmithKline) [11]. These p38α MAPK inhibitors displayed concentration-dependent inhibition of kinase activity when tested *in vitro*, with IC_50 _values (concentration of inhibitor required for 50% inhibition) well below the desired 1 μM *in vitro *inhibition activity sought before moving to *in vivo *studies. This landmark value is based on the finding that the *K*_*m *_values (concentration of substrate that gives half-maximal activity) for the two physiological substrates of signaling kinases, ATP and the respective endogenous protein substrate, range from 1–20 μM [39], and the assumption that a bioavailable and comparatively stable inhibitor should exhibit *in vivo *function if the target is valid. Approved kinase inhibitor drugs provide a precedent consistent with this guideline. Imatinib (Gleevec), the first example of these agents, has IC_50 _values of 0.1–0.35 μM for its kinase targets [40]. The p38α MAPK inhibitors in clinical development also showed selectivity of inhibition when examined by *in vitro *kinase inhibition screens that include pathway- and structurally-related protein kinases. Generally, there is some inhibition of p38β MAPK due to the close structural similarity between the p38α and p38β isoforms. However, p38δ MAPK and p38γ MAPK have key differences in the site targeted by many inhibitors designed by structure-assisted methods, allowing good selectivity against them [12,29,35-38,41,42].

Contemporary approaches to the design of selective p38α MAPK inhibitors take advantage of the extensive information available from high-resolution structures of p38 MAPK isoforms and complexes with inhibitors. Many p38α MAPK inhibitors are designed to exploit the small 'gatekeeper' amino acid that controls access to a hydrophobic binding pocket in the enzyme active site [37,38,41]. p38α and p38β MAPKs both have a threonine at the gatekeeper residue, 106, whereas p38γ and p38δ MAPKs have a significantly larger methionine present at the corresponding position. This enables the design of small molecules that can dock bulky constituents into the large hydrophobic pocket of the active site of the kinases with the smaller gatekeeper residue, that is, p38α and p38β MAPKs. Exploitation of this gatekeeper and hydrophobic pocket feature is combined with other aspects of the structure to create affinity and selectivity for p38α MAPK/p38β MAPK versus the other MAPK isoforms as well as other kinases. A recent example is the combination of the gatekeeper discrimination with another p38α MAPK/p38β MAPK structural feature, the potential for hydrogen bonding with the nearby peptide backbone segment that is conserved in the two kinases [37,38,41,42]. This is done by a vicinal arrangement of the two features in small molecules so that spacing and steric constraints within the targets are utilized.

## p38α MAPK as a drug discovery target for CNS disorders

Evidence from both clinical studies and preclinical animal models suggests proinflammatory cytokine overproduction as a potential driving force for pathology progression in CNS disorders [1,41,43-46]. Although the importance of p38α MAPK in the regulation of proinflammatory cytokine production in peripheral inflammatory disorders is well established, much less is known about its role in CNS inflammatory disorders. Glia and neuron cell culture studies demonstrated the importance of p38α MAPK activation for up-regulated cytokine production by stressed glia and provided a link between p38α MAPK and stressor-induced neuronal dysfunction *in vitro *[47-53]. In addition to glial p38α MAPK and its linkage in cell culture to cytokine increases in response to stressors, p38α MAPK is also expressed in neurons [54,55]. Neuronal p38α MAPK is considered a contributor to the phosphorylation and abnormal functioning of tau, a microtubule-associated protein found in neurons that correlates with the clinical pathology in AD and other dementias [56-59]. An interesting potential link between p38α MAPK activation in both glia and neurons comes from studies of co-cultures that showed microglia activation and release of IL-1β leading to increased neuronal tau phosphorylation [60]. Activation of p38α MAPK is also seen in brain tissue from AD transgenic mouse models [55,61,62]. Finally, clinical pathology results revealed the presence of activated p38α MAPK in brain samples from AD patients, with the activated p38α MAPK localized primarily to neurofibrillary tangles, neurons near neuritic amyloid plaques, and in glial cells [59,63-65].

**Figure 1:**
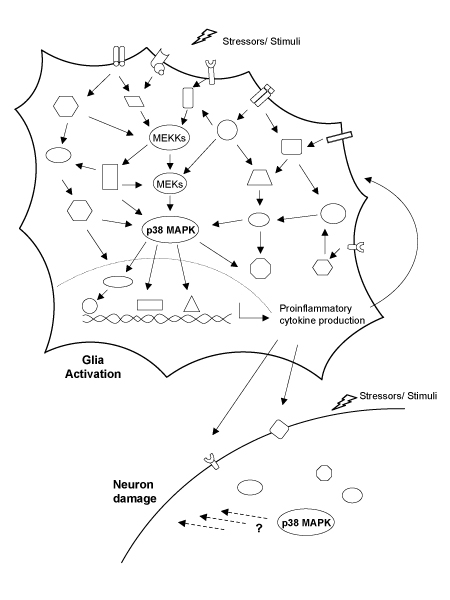
Diagrammatic outline of p38 mitogen-activated protein kinase (MAPK) pathways in glia and neurons. Disease-relevant stressors or stimuli can activate a variety of cross-talking and interacting signal transduction pathways, some of which can converge on activation of the p38 MAPK signaling cascades. For example, a typical p38 MAPK cascade consists of a three-tiered series of protein kinases: a MAPK (p38) and two upstream components (a MAPK kinase (MEK) and a MAPKK kinase (MEKK)) that activate the p38 MAPK by a series of activating phosphorylations. Activation of p38 MAPK and phosphorylation of its downstream substrates in activated glia (primarily microglia) can lead to up-regulation of proinflammatory cytokine production. Proinflammatory cytokines can act back on glia to stimulate multiple intracellular signaling pathways. Neurons can also respond to proinflammatory cytokine or other stressors/stimuli and activate neuronal p38 MAPK, culminating in neuron damage. The consequences of p38 MAPK activation in glia and neurons depend on the set of upstream signals, the isoform of p38 MAPK, the cell type, and the set of substrates that are stimulated.

An indication of the potential complexity of p38 MAPK involvement in the physiology and pathophysiology of glia and neurons is shown in Figure 1. This pictorial representation of the integrated and redundant aspects of intracellular signal transduction, mediated by protein phosphorylation pathways, indicates the currently unpredictable nature of how a particular pathway may be quantitatively involved in the response to a given stressor. The tissue context of a given cell type will add another variable. Due to the inherent limitations of removing glia and neurons from their tissue environment, it is critical that *in vivo *studies test explicit hypotheses about the quantitative importance of p38 MAPKs in specific pathophysiological progression mechanisms. This knowledge can provide a foundation for future therapeutic development campaigns.

An *in vivo *causative link between p38α MAPK and disease-relevant CNS pathophysiology has been provided by the use of small molecule p38 MAPK inhibitors in animal models of brain injury. For example, administration of a second-generation p38α MAPK inhibitor, SB239063, reduced infarct volume and attenuated neurological deficits in a rat model of focal ischemic brain injury [66,67]. Oral administration of a CNS-penetrant p38α MAPK inhibitor, MW01-2-069A-SRM, suppressed increases in hippocampal proinflammatory cytokine levels and the mechanistically associated synaptic marker protein loss in a mouse model of amyloid-beta (Aβ)-induced injury [41]. Consistent with the compound's positive effect on hippocampal synaptic marker protein levels was an attenuation of hippocampal-dependent behavioral deficits [41]. Such results indicate the potential for targeting p38α MAPK as a therapeutic approach in some CNS disorders and provide causative links between p38α MAPK, increased proinflammatory cytokine levels, and synaptic dysfunction.

**Figure 2:**
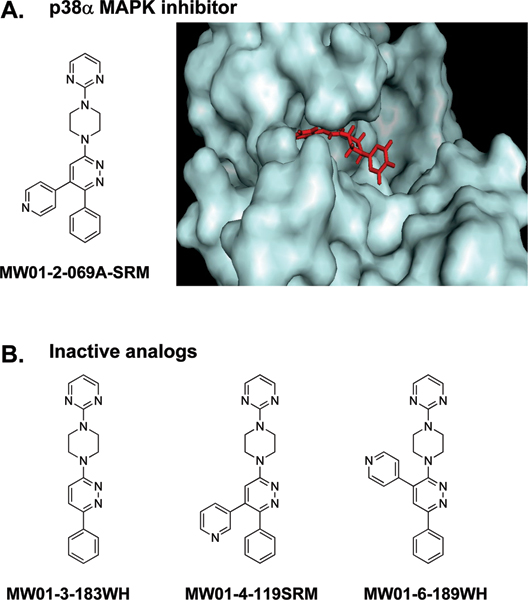
Strategy for development of a p38α mitogen-activated protein kinase (MAPK) inhibitor. **(A) **The p38α MAPK inhibitor MW01-2-069A-SRM was developed using a structure-assisted and computational modeling design strategy, along with consideration of compound molecular properties. The inhibitor is based on a 3-amino-6-phenyl pyridazine scaffold (MW01-3-183WH) found in other central nervous system (CNS)-active compounds [46,68]. A pyridinyl pharmacophore characteristic of many p38 MAPK inhibitors was added to the scaffold. Modeling forecasted that MW01-2-069A-SRM could be accommodated by the p38α MAPK structure. The smaller Thr106 gatekeeper residue allowed the phenyl ring of the compound to occupy a hydrophobic pocket while the nitrogen of the pyridine ring could make a critical H-bond interaction with the amide bond between Met109 and Gly110. These interactions are important for selectivity and affinity for the p38α MAPK isoform. MW01-2-069A-SRM was a p38α MAPK inhibitor, with an IC_50 _of approximately 0.8 μM, and was relatively selective for p38α MAPK; at 20 μM, the compound showed complete inhibition of p38α MAPK, partial inhibition of p38β MAPK, and no inhibition of p38δ MAPK, p38γ MAPK, or 40 other protein kinases [41]. **(B) **Validation of the design approach and modeling of predicted interactions was done by testing analogs that did not show the predicted interactions with the kinase. For example, the scaffold compound MW01-3-183WH, which lacks the pyridine ring, is inactive. The inactive analog MW01-4-199SRM has a pyridine nitrogen in a different structural orientation, which should compromise activity due to distance constraints and altered electronegativity. Another inactive analog, MW01-6-189WH, has an identical composition to MW01-2-069A-SRM, but has its pyridine ring in a different position on the pyridazine scaffold. These data show that the pyridine pharmacophore must be introduced adjacent to the phenyl group in the molecular context of the scaffold, as found in MW01-2-069A-SRM, to produce a p38α MAPK inhibitor with good affinity and selectivity.

The *in vivo *results with small molecule p38α MAPK inhibitors raise the possibility that appropriate dosing with bioavailable, blood brain barrier-penetrant, and non-toxic p38α MAPK inhibitors could generate a desired therapeutic outcome for CNS disorders. The mechanism could include a combined effect of attenuation of up-regulated proinflammatory cytokine production by activated glia and a potential neuroprotective effect on neuronal dysfunction. In terms of chronic neurodegenerative disorders like AD, compounds such as MW01-2-069A-SRM (Figure 2) have a number of appealing features [41]. The compound has molecular properties that are associated with successful human CNS therapeutics, and has good oral bioavailability and brain penetrance in rodents. For example, after oral administration to mice, the compound exhibited a peak brain:blood concentration ratio of approximately 0.7. Oral administration of MW01-2-069A-SRM at a low dose (2.5 mg/kg) after the start of exposure to the stressor (for example, toxic forms of human Aβ_1–42_) resulted in hippocampal levels of the proinflammatory cytokines TNFα and IL-1β, and the presynaptic marker protein synaptophysin, being indistinguishable from control levels measured weeks later [41]. These data provide evidence that the p38α MAPK pathway is quantitatively important in the Aβ-induced up-regulation of proinflammatory cytokines in the hippocampus, and that brain p38α MAPK should be considered as a credible molecular target in future drug development campaigns for AD and related neurodegenerative disorders.

## Conclusion

p38α MAPK is emerging as an attractive target for CNS disorders where increases in proinflammatory cytokines appear to play a role in disease progression. The development of orally bioavailable, brain-penetrant, CNS-active p38α MAPK inhibitors provides not only a valuable research tool for testing hypotheses about the role of p38α MAPK in CNS disorders, but also represents a foundation for future drug discovery efforts to develop potential neurodegenerative disease-modifying therapeutics that target this critical gene-regulating protein kinase.

## Abbreviations

Aβ: amyloid-beta; CNS: central nervous system; IL: interleukin; MAPK: mitogen-activated protein kinase; MSK1: mitogen and stress activated protein kinase 1; TNF: tumor necrosis factor.

## Competing interests

The authors declare that they have no competing interests.

## Authors' contributions

ASB coordinated literature searches and data analysis. LdA assisted with literature searches and figure preparation. DMW and LVE conceived of the project, and drafted the manuscript with the assistance of the other authors. All authors read and approved the final manuscript.
